# Amino acid-based enteral nutrition is effective for pediatric Crohn’s disease: a multicenter prospective study

**DOI:** 10.1093/gastro/goad072

**Published:** 2023-12-22

**Authors:** Qingfan Yang, Ting Zhang, Na Diao, Kang Chao, Huijun Shu, Jie Wu, Dexiu Guan, Li Wang, Xiwei Xu, Zhenghong Li, Xiang Gao

**Affiliations:** Department of Gastroenterology, The Sixth Affiliated Hospital, Sun Yat-sen University, Guangzhou, P. R. China; Guangdong Provincial Key Laboratory of Colorectal and Pelvic Floor Diseases, The Sixth Affiliated Hospital, Sun Yat-sen University, Guangzhou, P. R. China; Biomedical Innovation Center, The Sixth Affiliated Hospital, Sun Yat-sen University, Guangzhou, P. R. China; Department of Gastroenterology, Hepatology and Nutrition, Shanghai Children’s Hospital, School of Medicine, Shanghai Jiao Tong University, Shanghai, P.R. China; Department of Gastroenterology, The Sixth Affiliated Hospital, Sun Yat-sen University, Guangzhou, P. R. China; Guangdong Provincial Key Laboratory of Colorectal and Pelvic Floor Diseases, The Sixth Affiliated Hospital, Sun Yat-sen University, Guangzhou, P. R. China; Biomedical Innovation Center, The Sixth Affiliated Hospital, Sun Yat-sen University, Guangzhou, P. R. China; Department of Gastroenterology, The Sixth Affiliated Hospital, Sun Yat-sen University, Guangzhou, P. R. China; Guangdong Provincial Key Laboratory of Colorectal and Pelvic Floor Diseases, The Sixth Affiliated Hospital, Sun Yat-sen University, Guangzhou, P. R. China; Biomedical Innovation Center, The Sixth Affiliated Hospital, Sun Yat-sen University, Guangzhou, P. R. China; Department of Gastroenterology, Peking Union Medical College Hospital, Chinese Academy of Medical Sciences and Peking Union Medical College, Beijing, P. R. China; Department of Gastroenterology, Beijing Children’s Hospital, Capital Medical University, National Center for Children’s Health, Beijing, P. R. China; Department of Gastroenterology, Beijing Children’s Hospital, Capital Medical University, National Center for Children’s Health, Beijing, P. R. China; Department of Epidemiology and Biostatistics, Institute of Basic Medical Sciences Chinese Academy of Medical Sciences, School of Basic Medicine Peking Union Medical College, Beijing, P. R. China; Department of Gastroenterology, Beijing Children’s Hospital, Capital Medical University, National Center for Children’s Health, Beijing, P. R. China; Department of Pediatrics, State Key Laboratory of Complex Severe and Rare Diseases, Peking Union Medical College Hospital, Chinese Academy of Medical Science and Peking Union Medical College, Beijing, P. R. China; Department of Gastroenterology, The Sixth Affiliated Hospital, Sun Yat-sen University, Guangzhou, P. R. China; Guangdong Provincial Key Laboratory of Colorectal and Pelvic Floor Diseases, The Sixth Affiliated Hospital, Sun Yat-sen University, Guangzhou, P. R. China; Biomedical Innovation Center, The Sixth Affiliated Hospital, Sun Yat-sen University, Guangzhou, P. R. China

**Keywords:** pediatric Crohn’s disease, exclusive enteral nutrition, amino acid-based enteral formula, prospective study, effectiveness, nutrition improvement

## Abstract

**Background:**

Exclusive enteral nutrition (EEN) therapy effectively induces remission in pediatric Crohn’s disease (CD). However, this may depend on the type of enteral formula used. Moreover, data on the efficacy of amino acid-based EEN are limited. Thus, we aimed to prospectively evaluate the efficacy of amino acid-based formulas for EEN in pediatric patients with active CD.

**Methods:**

Patients with active CD aged between 6 and 17 years were recruited into this prospective study from four hospitals in China between March 2019 and December 2021. Patients received EEN for 8 weeks. Inflammatory and nutrition-associated indices were evaluated at 0, 4, and 8 weeks after treatment. Paired *t*-tests and Wilcoxon signed-rank tests were used to compare continuous and categorical variables before and after intervention, respectively.

**Results:**

Twenty-four patients were included in the analysis. After an 8-week intervention period, the CD activity index significantly decreased (26.3 ± 12.2 vs 7.1 ± 8.3, *P *<* *0.001). Most patients (66.7%) achieved complete clinical remission. Among the 22 patients who had ulcers and erosions diagnosed endoscopically at baseline, 10 (45.5%) achieved complete mucosal healing. The degree of thickening of the intestinal wall was significantly reduced after EEN intervention, with a transmural healing rate of 42.9%. Furthermore, the serum inflammatory markers decreased and there was a significant improvement in the nutrition-related indices (*P *<* *0.05). There were no severe adverse effects.

**Conclusions:**

Amino acid-based EEN is effective and safe for treating pediatric-onset CD. Studies with larger sample sizes and mechanistic and follow-up studies are required to further validate these findings.

## Introduction

Crohn’s disease (CD) is a chronic inflammatory bowel disease that affects the entire gastrointestinal tract. The exact cause of CD is still unknown but it is characterized by transmural inflammation of the intestinal wall, which can result in the formation of deep ulcerations, abscesses, fistulae, and strictures [1, 2]. Typical symptoms of CD include diarrhea, abdominal pain, rectal bleeding, fever, weight loss, exhaustion, anemia, recurring fistulas, intestinal obstruction, and extra-intestinal manifestations [3, 4]. Approximately 25% of new cases are diagnosed in children and young adults [5]. Pediatric CD incidence has increased dramatically over the past decade, ranging from 2.5 to 11.4 cases per 100,000 [6–8]. Its course is more severe in children than in adults, with ∼50% of children requiring surgery within 10 years of diagnosis [9–11]. Most children with CD have a history of malnutrition [12], which causes micronutrient deficiency, growth retardation, delayed puberty, and disability [13]. Therefore, early intervention and management of intestinal inflammation are critical in pediatric patients.

Current induction therapy guidelines for pediatric CD include corticosteroids, antitumor necrosis factor-α (TNF-α), and exclusive enteral nutrition (EEN) [8]. Nutritional intervention is essential due to the high malnutrition rates, growth deficiency, and delayed pubertal development among children with CD. The GROWTH-CD study demonstrated that EEN more effectively induced remission than corticosteroids and had other benefits, including improved growth [14]. Studies also suggest that the proportion of clinical and endoscopic remission in EEN (88.57% and 68.75%) was similar to those in an anti-TNF-α agent (infliximab, 73.91% and 80.77%) after induction therapy [15]. Overall, EEN is clinically efficacious and is associated with improved mucosal healing (MH), linear growth, and bone health during induction treatment in pediatric CD [16, 17].

However, different nutritional formulas, study populations, and cultures may influence the effectiveness of EEN in active CD. The enteral formulas used in CD can be divided into three types according to the protein composition: polymeric-, peptide-, and amino acid-based formulas. A recent meta-analysis included comprehensive studies investigating the potential effects of EEN in children with CD [18]. Among the 46 studies found, most used polymeric-based enteral formulas and only a few chose amino acid-based formulas. Furthermore, all studies with an amino acid-based formula were of Western origin. Thus, studies on the effect of amino acid-based enteral nutrition in the pediatric population are few, particularly in Asian children.

Therefore, in this study, we aimed to prospectively analyse the data of children with CD enrolled from multiple centers in China and administered an 8-week course of EEN with an amino acid-based enteral formula.

## Materials and methods

### Patients and study design

**Figure 1.**
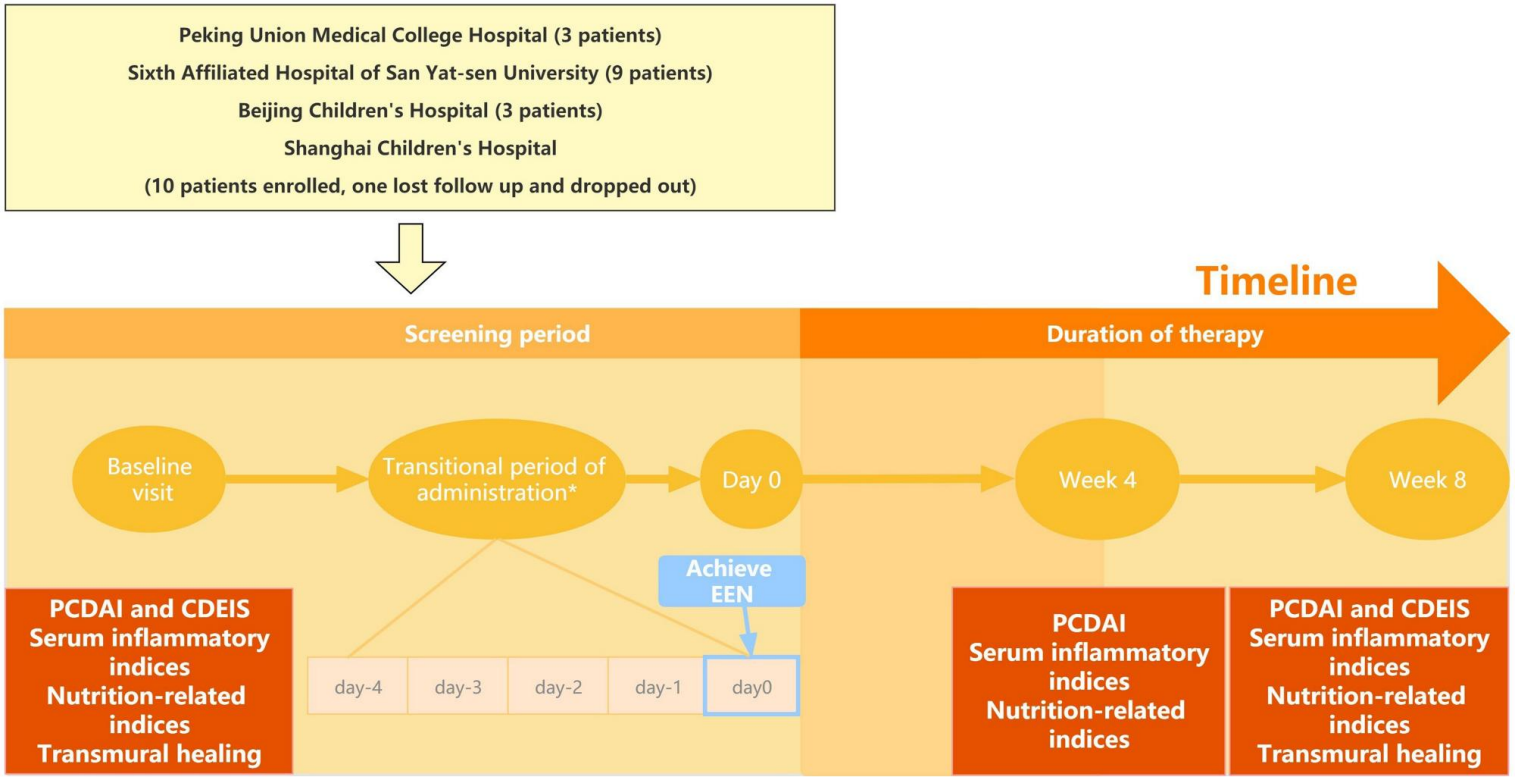
Study design. Patients were followed from the baseline visit to Week 8 at specific time points with clinical assessment. The initial dosage was approximately one-eighth of the daily requirement and the concentration was halved (0.5 kcal/mL). The concentration and dosage were gradually increased over Days 4–10 to reach the standard dosage (adaptation phase). The infusion speed was also increased in a stepwise manner so that patients received 20 mL/h by the end of the adaptation phase (transitional period of administration last from 4 to 10 days). EEN = exclusive enteral nutrition, PCDAI = Pediatric Crohn’s Disease Activity Index, CDEIS = Crohn’s Disease Endoscopic Index of Severity.

This multicenter, prospective study was conducted between March 2019 and December 2021, and patients were recruited from four large hospitals in Guangdong, Beijing, and Shanghai (Figure 1). The study adhered to the ethical principles of the Declaration of Helsinki and Good Clinical Practice guidelines. Patients or their parents/legal representatives provided written informed consent. The study protocol was approved by the Institutional Review Boards of the four hospitals. This trial was registered with the Chinese Clinical Trial Registry (registration number 1900026979).

Patients who met the following criteria were consecutively recruited: (i) children aged 6–17 years; (ii) diagnosis of CD according to established international criteria [19]; (iii) a baseline Pediatric Crohn’s Disease Activity Index (PCDAI) of ≥10, demonstrating active luminal disease [20].

We excluded patients with (i) recent antibiotic treatment (within 2 weeks of study commencement); (ii) CD-related surgery or medications (i.e. thiopurines and biological agents) within 1 month of study commencement; and (iii) previously diagnosed short bowel syndrome, high-flow fistula, complete intestinal obstruction, cancer, or immune system disease.

### Intervention

Patients received the elemental enteral formula Elental^®^ (EA Pharma Co., Ltd., Tokyo, Japan) for 8 weeks as applied by previous studies [8, 18]. This formula is composed of amino acids, low amounts of fat, vitamins, trace elements, and dextrin as a major energy source (Supplementary Table 1). The calorie density of this formula was 1 kcal/mL, with an osmolarity of 610 mOsm/L. The elemental formula was continuously infused through a nasogastric tube using an infusion pump. The initial dosage administered was approximately one-eighth of the daily requirement and the concentration was halved (0.5 kcal/mL). The concentration and dosage were gradually increased over Days 4–10 to reach the standard dosage (adaptation phase). Moreover, the infusion speed was increased in a stepwise manner so that patients were receiving 20 mL/h by the end of the adaptation phase (Figure 1). The amount of daily caloric needs for each individual is mainly based on age and body weight. Infants <1 year old need ∼110 kcal/kg/day; 10 kcal is subtracted every 3 years to obtain 60 kcal/kg/day at 15 years and 25–30 kcal/kg/day for adults [21]. The compliance rate is expected to be 90%, as reported in a previous study [22]. Patients who used mesalazine with no obvious improvement in disease and without any adverse effects before inclusion were allowed to continue the usage of mesalazine at a dosage not exceeding 2 g/day after the full evaluation and with the consent of the investigator. Patients who failed steroid induction therapy had their steroid dosage reduced to 20 mg/day before enrollment. The use of other enteral nutritional formulas, biological agents, antibiotics, and other foods except water was not allowed.

### Clinical, endoscopic, and blood tests

The participants’ demographics and clinical characteristics were collected. The clinical classification of CD was assessed using the Montreal classification [23]. Compliance, adverse effects, general well-being, and clinical symptoms were monitored and recorded throughout the study. PCDAI score, STRONGkids score (screening tool for risk on nutritional status and growth), height, body weight, and body mass index (BMI) were recorded. White blood cell count, serum albumin, hemoglobin, serum prealbumin, lymphocyte count, trace elements, high-sensitivity C-reactive protein, platelets (PLT), and erythrocyte sedimentation rates were measured at baseline and at Weeks 4 and 8. Colon endoscopy, computed tomography enterography and/or magnetic resonance enteroclysis, and intestinal ultrasound were performed at baseline and Week 8 to evaluate intestinal inflammation (Figure 1).

### Outcome measures

Primary outcomes were clinical remission and MH rate. PCDAI was used to evaluate clinical remission, defined as PCDAI of <10. However, PCDAI scores of 10–27.5, 30–37.5, and ≥40 represent mild, moderate, and severe disease, respectively [20]. MH was evaluated using the Crohn’s Disease Endoscopic Index of Severity (CDEIS) [24]. CDEIS scores of 3–8, 9–11, and ≥12 represent mild, moderate, and severe endoscopic activity, respectively. A score of <3 indicates MH.

Secondary outcomes included improvement in nutritional status, changes in serum inflammatory markers, transmural healing (TH) rate, and the safety of elemental enteral formula. STRONGkids score, nutrition-related laboratory markers, and changes in height and BMI using z-scores based on World Health Organization standards were used to evaluate patients’ nutritional status. TH was defined as an intestinal wall thickness not exceeding 3 mm, as evaluated by using intestinal ultrasound.

### Statistical analysis

The expected clinical remission rate of EEN therapy with an amino acid-based formula was 80% according to clinical experience and prior reports [17]. An acceptable 95% confidence interval width (two-sided) of 30% was set and the calculated sample size was 26. Assuming a dropout rate of 20%, 31 patients were expected to be included. Quantitative data are expressed as mean ± standard deviation or as medians with interquartile ranges (IQRs). The height-for-age z-score (HAZ) and BMI-for-age z-score (BAZ) for each child before and after treatment were calculated using World Health Organization AuthorPlus software. Differences between the median values were compared using the Mann–Whitney *U* test. Paired *t*-tests and Wilcoxon signed-rank tests compared continuous and categorical variables before and after EEN intervention, respectively; *P *<* *0.05 was considered statistically significant. All measurements were analysed by the Department of Epidemiology and Biostatistics of the Institute of Basic Medical Sciences, Chinese Academy of Medical Sciences, and Peking Union Medical College using SAS (version 9.4; SAS Institute, Cary, NC, USA).

## Results

### Patient characteristics

We enrolled 25 patients in the study from the four hospitals. One patient was lost to follow-up; therefore, data from 24 patients who completed the study were analysed (Figure 1). Patients were aged 14.1 ± 2.2 years and 15 (62.5%) were male. Eighteen (75.0%) patients were classified as having ileocolon CD and 22 (91.6%) had non-stenotic and non-penetrating behavior (Table 1).

**Table 1.** Demographic characteristics of 24 patients with Crohn’s disease enrolled in the study

Characteristic	Value
Male, *n* (%)	15 (62.5%)
Age, years, mean *±* SD	14.1 ± 2.2
Disease course, months, mean *±* SD	13.9 ± 11.0
Family history	0 (0)
Behavior	
B1	22 (91.6%)
B2	1 (4.2%)
B3	1(4.2%)
Location	
L1	3 (12.5%)
L2	0 (0)
L3	18 (75.0%)
L1+L4	1 (4.2%)
L3+L4	2 (8.3%)
Previous surgical history	
Appendectomy	2 (8.3%)
Perianal surgery	5 (20.8%)
Other gastrointestinal surgery	0 (0)
Previous perianal disease	5 (20.8%)
Intestinal fistula/abscess	1 (4.2%)
Medication history	
Steroids	1 (4.2%)
Mesalazine	2 (8.3%)
Anti-TNF-α agent	5 (20.8%)
Drugs used during EEN	
Mesalazine	2 (8.3%)
Steroid	0 (0)

B1 = non-stenotic and non-penetrating behavior, B2 = stenotic behavior, B3 = penetrating behavior, L1 = terminal ileum, L2 = colon, L3 = ileum colon, L4 = upper gastrointestinal tract, TNF = tumor necrosis factor, EEN = exclusive enteral nutrition, SD = standard deviation.

### Primary outcomes

#### Clinical remission rate

**Figure 2.**
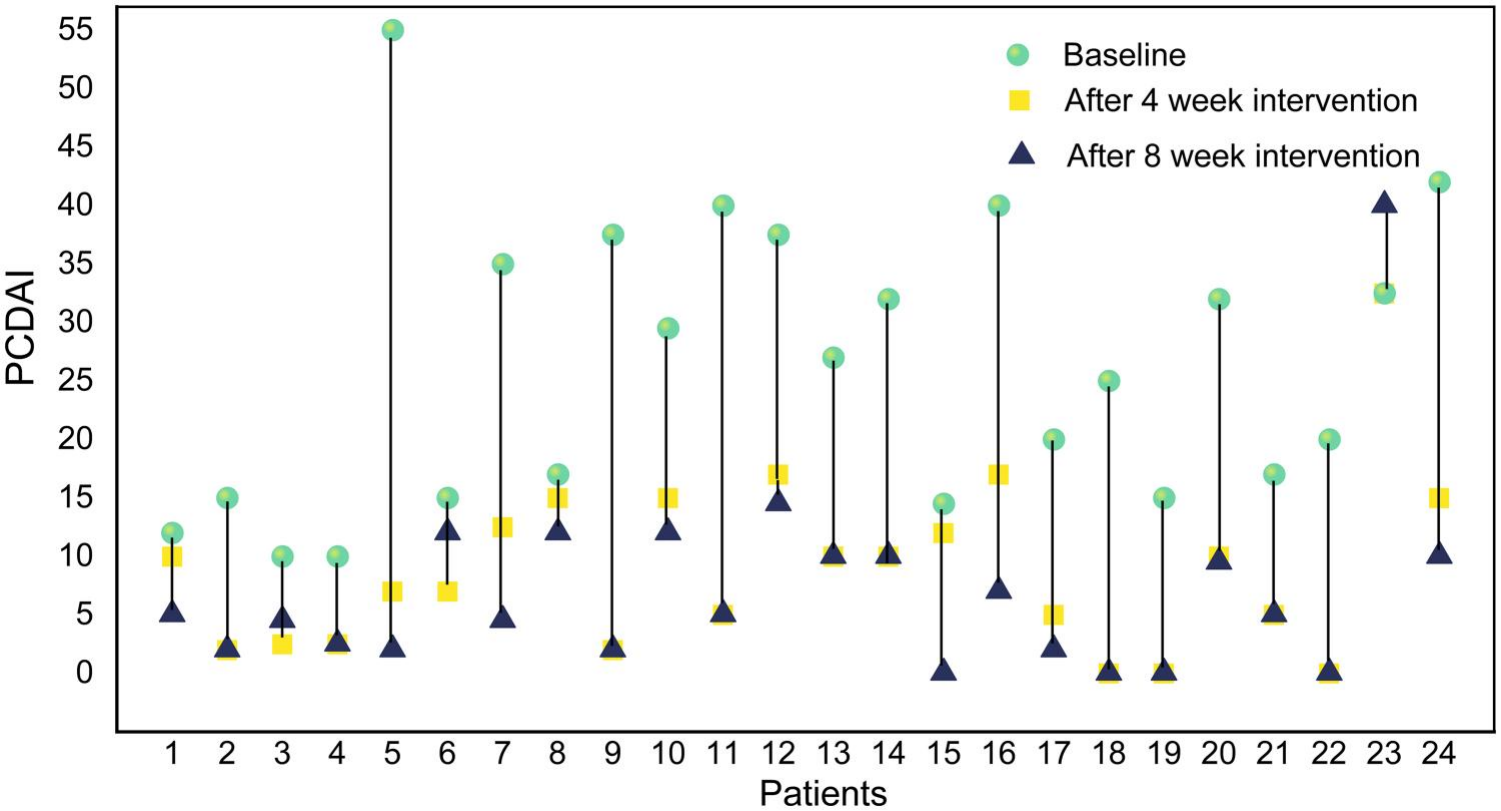
Changes in PCDAI in each patient after EEN intervention. PCDAI score significantly reduced after EEN. EEN = exclusive enteral nutrition, PCDAI = Pediatric Crohn’s Disease Activity Index.

All patients had an active CD at baseline. According to the PCDAI, 13 (54.2%), 7 (29.1%), and 4 (16.7%) patients had a mild, moderate, and severe active disease, respectively. Furthermore, 12 patients (50.0%) achieved clinical remission after 4 weeks of EEN therapy. By the eighth week, 16 (66.7%) patients achieved clinical remission (Figure 2). Compared with baseline (26.3 ± 12.2), PCDAI scores decreased significantly after 4 (8.9 ± 7.5) and 8 (7.1 ± 8.3) weeks of EEN (*P *<* *0.001; Table 2).

**Table 2.** Changes in PCDAI after EEN intervention

Time	PCDAI	*P*-value	Clinical remission rate (%)
Baseline	26.3 ± 12.2	–	–
After 4-week EEN	8.9 ± 7.5	<0.001	50.0%
After 8-week EEN	7.1 ± 8.3	<0.001	66.7%

PCDAI = Pediatric Crohn’s Disease Activity Index, EEN = exclusive enteral nutrition.

#### MH rate

**Figure 3.**
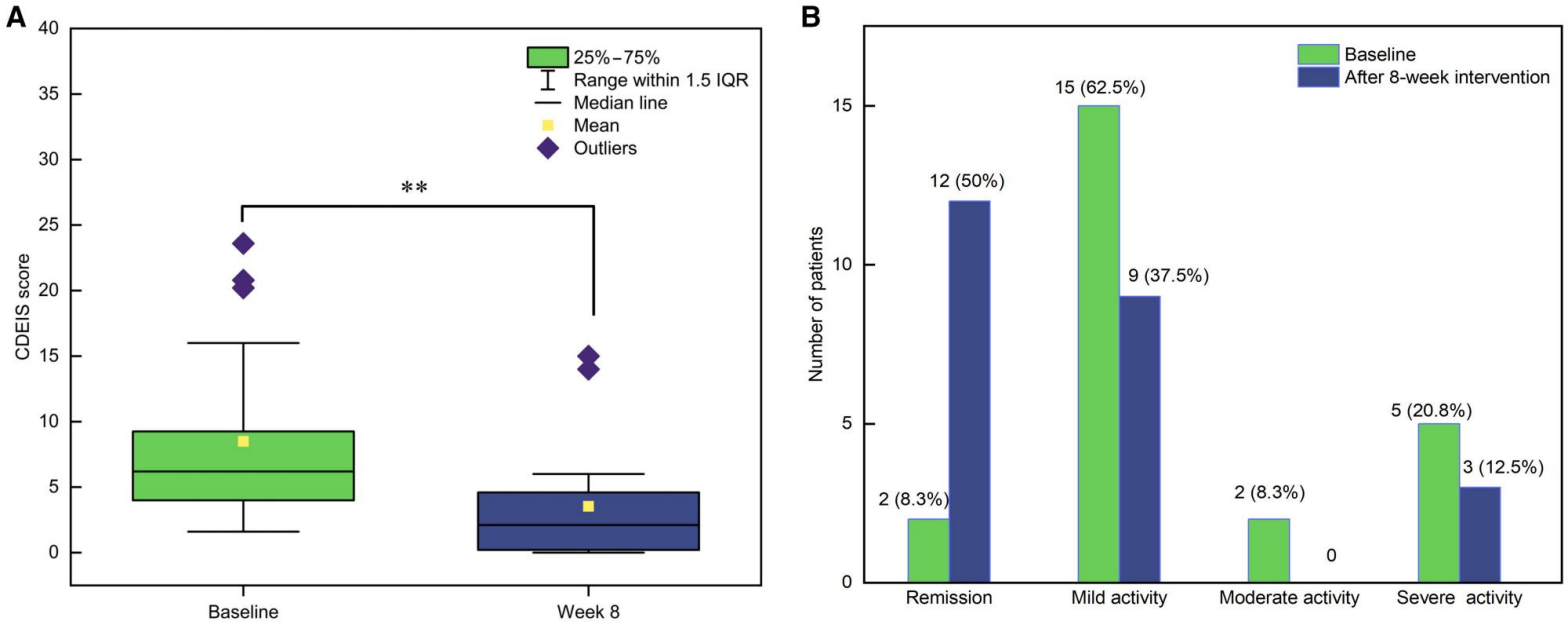
Changes in CDEIS after EEN intervention. (A) Changes in CDEIS score after EEN intervention. CDEIS score significantly reduced after EEN (*P *<* *0.001). (B) Changes in disease activity under endoscopy after EEN intervention. Disease activity showed an obvious downward trend (Wilcoxon signed-rank test, *P *=* *0.009). **P <* *0.001; CDEIS = Crohn’s Disease Endoscopic Index of Severity, IQR = interquartile range, EEN = exclusive enteral nutrition.

**Figure 4.**
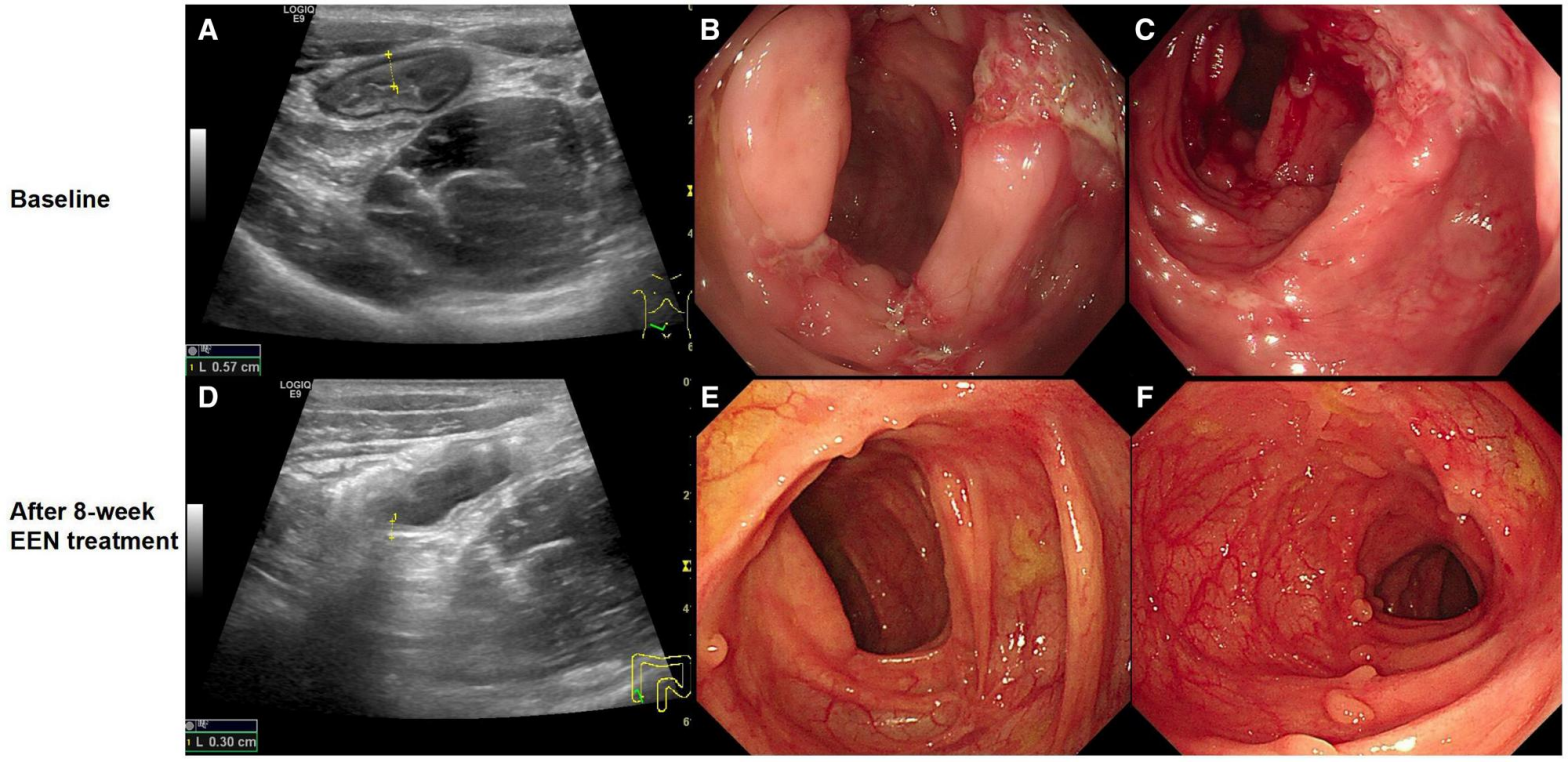
Patients achieved transmural healing and mucosal healing after EEN. Before EEN, the bowel wall was thickened (5.7 mm) in a patient enrolled in the study (A), which was reduced to 3 mm after 8-week EEN (D), determined by using a bowel sonography examination. Irregular ulcers were seen in the ileocecal valve (B) and ascending colon (C) under colonoscopy in the pediatric patients with CD; the ulcers disappeared (**E** and **F**) and MH was achieved after 8 weeks of EEN. CD = Crohn’s disease, EEN = exclusive enteral nutrition, MH = mucosal healing.

Twenty-two (91.6%) patients presented with ulcers and erosions on endoscopy and CDEIS of >3 at baseline. Following EEN, lesions disappeared in 10 patients (CDEIS < 3), signifying a MH rate of 45.5%. According to baseline CDEIS scores, 2 (8.3%) patients were in MH, and 15 (62.6%), 2 (8.3%), and 5 (20.8%) had mild, moderate, and severe active disease, respectively. At 8 weeks, 12 (50.0%) patients had MH, and 9 (37.5%) and 3 (12.5%) had mild and severe active disease, respectively. No patient had moderately active disease. CDEIS score significantly reduced after EEN [6.2 (4.0–9.1) at baseline vs 2.1 (0.2–4.5) after 8 weeks of EEN, *P *<* *0.001]. Disease activity showed an obvious downward trend (*P *=* *0.009; Figures 3 and 4).

### Secondary outcomes

#### Assessment of inflammatory parameters and TH

High-sensitivity C-reactive protein decreased from 13.0 (4.7, 26.6) mg/L at baseline to 5.0 (0.5, 5.0) mg/L after treatment (*P *=* *0.012). Furthermore, PLT decreased from (370.7 ± 93.7) × 10^9^/L before treatment to (319.4 ± 70.5) × 10^9^/L after treatment (*P *=* *0.001). The erythrocyte sedimentation rate decreased from 39.0 (20.0, 58.5) mm/h before treatment to 18.5 (5.8, 24.2) mm/h at Week 8 (*P *=* *0.002) (Table 3).

**Table 3.** Inflammatory and nutrition-associated indices before and after EEN

Index	Before EEN	After 8-week EEN	*P*-value
High-sensitivity C-reactive protein, mg/L, median (IQR)	13.0 (4.7, 26.6)	5.0 (0.5, 5.0)	0.012
PLT, × 10^9^/L, mean ± SD	370.7 ± 93.7	319.4 ± 70.5	0.001
Erythrocyte sedimentation rate, mm/h, median (IQR)	39.0 (20.0, 58.5)	18.5 (5.8, 24.2)	0.002
Albumin, g/L, mean ± SD	37.0 ± 4.9	40.4 ± 3.9	< 0.001
Hemoglobin, g/L, mean ± SD	114.5 ± 12.9	123.9 ± 14.6	0.001
STRONGkids score, mean ± SD	3.6 ± 1.4	2.1 ± 1.2	< 0.001
HAZ, median (IQR)	–0.3 (–0.6, 0.8)	–0.2 (–0.6, 0.7)	0.626
BAZ, median (IQR)	–1.5 (–2.5, –0.2)	–1.1 (–1.9, –0.3)	0.008

BAZ = body mass index-for-age z-score, EEN = exclusive enteral nutrition, HAZ = height-for-age z-score, IQR = interquartile range, PLT = platelet, STRONGkids score = screening tool for risk on nutritional status and growth.

Fifteen patients underwent a paired bowel sonography examination at the start and end of the study following overnight fasting. At baseline, 14 patients displayed a thickening of the bowel wall. After EEN, six (42.9%) patients achieved TH, with a bowel wall thickness of ≤3 mm. Intestinal wall thickness decreased after the enteral formula intervention compared with that at baseline (3.93 ± 1.67 vs 6.13 ± 2.34 mm; *P *<* *0.001; Figure 4). All patients who achieved TH exhibited MH under endoscopy. Two out of the eight patients who did not achieve TH achieved MH.

#### Nutrition improvement after amino acid-based enteral formula intervention

**Figure 5.**
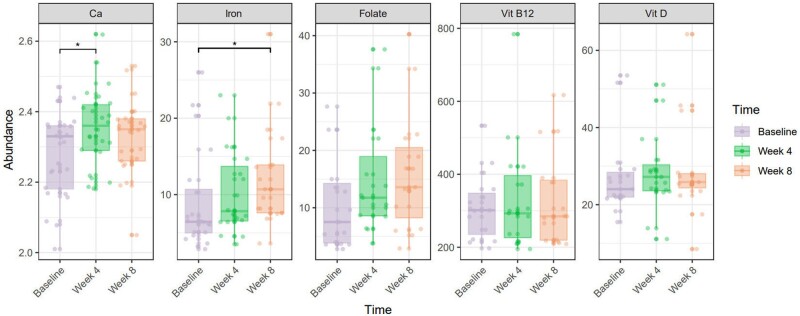
Changes in the amount of trace elements after EEN intervention. EEN = exclusive enteral nutrition, Ca = calcium.

According to the STRONGkids assessment, 50% of patients had moderate and 50% had a high nutritional risk at baseline. After the 8-week intervention, 4 (16.7%), 19 (79.2%), and 1 (4.2%) patient(s) exhibited low, moderate, and high nutritional risk, respectively, reflecting a significant decrease in risk (*P *=* *0.0001). The STRONGkids score decreased significantly from 3.6 ± 1.4 at baseline to 2.1 ± 1.2 at Week 8 (*P *<* *0.001; Table 3). Furthermore, nutrition-related indices, such as serum albumin and hemoglobin, significantly improved (*P *<* *0.05). Changes in trace elements were detected in blood samples. At baseline, levels of calcium, iron, vitamin D, and folate were below normal at 20.8%, 41.7%, 25%, and 16.7%, respectively. After EEN, these levels improved, with a notable increase in iron [6.5 (5.1. 11.1) vs 8.2 (7.2, 13.8) mmol/L, *P *=* *0.031; Figure 5].

Regarding patient growth, HAZ ranged from –1.9 to 2.5 [median (IQR) –0.3 (–0.6, 0.8)] before EEN and no patient had growth retardation. BAZ ranged from –6.0 to 2.0 [median (IQR) –1.5 (–2.5, –0.2)] before EEN and 17 (70.8%) patients were within the normal range (Table 3). The mean z-score values for each of the growth parameters were compared at baseline and Week 8; there was a significant difference in BAZ [–1.5 (–2.5, –0.2) vs –1.1 (–1.9, –0.3), *P *=* *0.008], but no significant difference in HAZ scores (*P *=* *0.626).

#### Adverse effects

Six adverse events were reported in three patients. One patient reported sinus arrhythmia and sinus bradycardia at a single time point. Another patient reported sinus arrhythmia on two occasions. Fatty liver was reported by another patient who also experienced a bout of diarrhea that subsided after a short period.

## Discussion

This study supports the clinical efficacy of EEN with an amino acid-based elemental formula in children with CD. Clinical remission was achieved in 66.7% of patients and 45.5% achieved complete MH with a significant decline in PCDAI and CDEIS scores after treatment. Furthermore, TH, nutritional status, and growth improved with minimal adverse effects.

The primary outcomes investigated in this study were clinical remission and MH rate. The clinical remission rate was 50.0% after 4 weeks of EEN and 66.7% at the end of treatment with the amino acid-based formula. Inflammatory markers, such as high-sensitivity C-reactive protein, erythrocyte sedimentation rate, and PLT, also decreased. Most studies involving EEN treatment for children with CD were analysed with polymeric-based formulas. Moreover, most studies based on amino acid-based enteral nutrition were retrospective. Consequently, there are limited prospective data investigating the benefits of amino acid-based formulas in Asian children. Therefore, this study significantly enriches the knowledge in this field. Ludvigsson *et al.* [25] prospectively examined 16 children with CD receiving EEN with an amino acid-based formula (Elemental 028 Extra); 69% of the patients achieved clinical remission after 6 weeks. Similarly, Johnson *et al.* [26] reported that 16 children with CD receiving amino acid-based EEN (Elemental 028 Extra) for 6 weeks had a remission rate of 42% and decreased inflammatory markers, including PLT and erythrocyte sedimentation rate. The clinical remission rate observed in our study was similar to that reported in the two aforementioned prospective studies. Another prospective single-center pilot study examined the impact of elemental diet therapy with Elental^®^ in adults with CD and reported a clinical remission rate of 71% after 4 weeks, which is higher than ours [27]. Additionally, we found higher clinical remission rates after 8 weeks of EEN than after 4 weeks, suggesting that 8-week therapy should be recommended in children. Overall, our study demonstrated that amino acid-based enteral nutrition effectively induced remission in children with CD.

Although MH is considered an objective treatment goal for CD, there are limited high-quality data on its response to EEN in pediatric patients. In our study, we observed a complete MH rate of 45.5% after 8 weeks of intervention, with a concomitant decrease in the CDEIS score. The prospective studies by Ludvigsson *et al*. [25] and Johnson *et al*. [26] did not examine the impact of amino acid-based EEN on MH. A prospective study performed in adults with CD using Elental^®^ reported a MH rate of 44% in the terminal ileum and 39% in the large bowel after a 4-week intervention, using a different evaluation method than ours [27]. A randomized–controlled study of patients with CD demonstrated that more patients achieved MH following 10 weeks of polymeric EEN compared with patients receiving corticosteroids (74% vs 33%, *P *<* *0.05) [28]. Fell *et al.* [29] demonstrated complete MH in 79% of pediatric patients with CD after 8 weeks of polymeric formula-based EEN. Grover *et al.* [30] demonstrated an improvement in endoscopy assessment following a 6-week course of EEN therapy using a polymeric-based formula. MH was achieved in 43% of pediatric patients determined by the simple endoscopic score for CD. Another study demonstrated a complete MH in 33% of patients who received ≥6 weeks of EEN using the simple endoscopic score for CD without detailing the enteral formula type [16]. Yao *et al.* [15] retrospectively reported a MH rate in 73.3% and 80.8% of pediatric patients in the infliximab- and polymeric-based EEN groups, respectively (*P *=* *0.374). As mentioned above, most pediatric studies reported the MH rate with a polymeric-based formula, while data based on an amino acid enteral formula are limited. Our study provides important evidence of the benefit of this therapy on MH in children with CD prospectively.

Objective TH has recently gained attention as a novel therapeutic target for CD; however, data on the TH rate with amino acid-based EEN treatment are limited. Grover *et al.* [16] reported that 3 of 14 (21%) adult patients achieved complete transmural remission of ileal CD following a 6-week intervention with a polymeric-based formula determined by magnetic resonance enteroclysis evaluation. Similarly, a prospective observational study by Chen *et al.* [31] found that 5 of 29 (17%) adult patients achieved TH with a significant reduction in bowel wall thickness assessed by bowel sonography following 50–212 days of polymeric formula-based EEN therapy. In the present study, our findings revealed a 42.9% TH rate by bowel sonography examination and all patients with TH achieved complete MH. The reported rate of TH ranges from 14% to 68%, mainly based on the use of biologics in CD treatment [32]. Our study showed that 8 weeks of EEN therapy with an amino acid-based formula could successfully induce TH in children with CD, likely with a comparable TH to that of biologics.

When treating pediatric CD, it is important to maintain growth, minimize adverse effects, and manage symptoms. In this study, we observed improved nutritional status of patients following treatment with an amino acid-based enteral formula. Nutrition-related indices, such as serum albumin and hemoglobin, were significantly increased following therapy. Before EEN, the amount of trace elements, such as iron and Ca, were high. However, after our treatment, these high amounts were reversed. Notably, serum iron levels significantly improved despite the short intervention period. The z-scores of height and BMI also improved.

No significant adverse events were observed during EEN treatment in our study. However, sinus arrhythmia or bradycardia was reported in two patients, which were thought to be unrelated to EEN intervention. Fatty liver and a short bout of diarrhea were reported in one patient, which may have been related to the use of an amino acid-based formula. A previous study using Elental^®^ to induce CD remission in adult patients reported side effects such as diarrhea, abdominal distension, and abdominal colic [27]. Overall, EEN with the amino acid enteral formula is safe for pediatric CD.

We comprehensively analysed the effect of EEN with an amino acid-based enteral formula on children with CD. However, the difference in impact between this formula and polymeric-based enteral nutrition in treating CD is still unclear. Previous studies inclined a similar effect of the two kinds of enteral formula on inducing clinical remission in pediatric CD [17, 18]. However, the exact efficacy of these types of enteral formula on patients with different disease behaviors or locations has not been systematically reported. Similarly to ours, previous studies reported an MH rate of 43%–80.8% with polymeric-based enteral nutrition in pediatric CD [15, 16, 29, 30]. The MH rate in the present study was 45.5% after an 8-week intervention. Previous studies reported a TH rate of 17% and 21% with polymeric-based enteral nutrition in pediatric or adult CD [30, 31]. The TH rate was 42.9% in our study. However, it is not conclusive whether the impact on MH and TH are different. We observed an improvement in the nutrition-related indices after amino acid-based EEN in this study, and EEN with a polymeric-based formula reportedly improved nutritional indices, lean muscle mass, weight, height, and micronutrients in patients with CD [33]. As for adverse events, a recent meta-analysis suggests no significant difference between polymeric- and amino acid-based formulas [17]. In total, comparative studies evaluating the effects of the two formulas are limited. More research is required to further understand how they differ in terms of MH, TH, nutritional enhancement, and safety.

Our study had a few limitations. First, the sample size was small and it was difficult to conduct a blinded study owing to the characteristics of EEN. The study design did not include patients receiving polymeric-based enteral nutrition for effect comparison. Therefore, future studies with larger sample sizes, better designs, refined inclusion criteria, and knowledge of possible confounders are needed to confirm the observations reported in this study. Data justice should also be fully considered to ensure the safety, accuracy, and fairness of the study. Second, we only discussed the short-term effect of EEN with the amino acid-based formula. Studies investigating the long-term effects are required. Third, we only analysed the clinical effects of our formula. The mechanisms by which it improves CD have not been fully elucidated. It may mediate gut microbial and metabolic changes that are associated with remission in children with CD [34, 35]; however, most mechanistic studies use polymeric- or peptide-based formulas and the modulatory role of the elemental formula in CD requires further study.

## Conclusions

EEN intervention with an amino acid-based formula was found to be effective in inducing clinical remission and MH in children with CD. During an 8-week therapy period, clinical activity scores, biochemical markers of disease activity, and growth parameters improved. Furthermore, EEN was well tolerated, with no serious adverse events. The findings of this study demonstrate that amino acid-based EEN is safe and efficacious in the treatment of pediatric-onset CD. Further studies with large sample sizes and better study designs to validate the findings are needed.

## Supplementary Material

goad072_Supplementary_Data
