# Correction to: Amino acid-based enteral nutrition is effective for pediatric Crohn’s disease: a multicenter prospective study

**DOI:** 10.1093/gastro/goae107

**Published:** 2024-12-28

This is a correction to: Qingfan Yang, Ting Zhang, Na Diao, Kang Chao, Huijun Shu, Jie Wu, Dexiu Guan, Li Wang, Xiwei Xu, Zhenghong Li, Xiang Gao, Amino acid-based enteral nutrition is effective for pediatric Crohn’s disease: a multicenter prospective study, *Gastroenterology Report*, Volume 12, 2024, https://doi.org/10.1093/gastro/goad072

In the originally published version of the manuscript, in section **Material and methods**, under heading **Intervention**, in the first sentence, the manufacturer information was erroneous. This should read: “Patients received the elemental enteral formula Elental^®^ (EA Pharma Co., Ltd., Tokyo, Japan)[…]” instead of: “Patients received the elemental enteral formula Elental^®^ (Ajinomoto; Morishita-Russel, Tokyo, Japan)[…].” The end of the third sentence should read: “[…]with an osmolarity of 610 mOsm/L.” instead of: “[…]with an osmolarity of 760 mOsm/L.”

The emendations have been made to the article.

