# Supplementary material for: Amino acid-based enteral nutrition is effective for pediatric Crohn’s disease: a multicenter prospective study
Source: Gastroenterol Rep (Oxf). 2023 Dec 22;12:goad072. doi: 10.1093/gastro/goad072 (PMC10746840; doi:10.1093/gastro/goad072)
Supplement: goad072_Supplementary_Data [file goad072_supplementary_data.docx]

**Supplementary Table 1.** Composition of Elental®

| Nutrients | 100 g (375 kcal) |
| --- | --- |
| Amino acids | 17.6 g (66 kcal) |
| L-Isoleucine | 803 mg |
| L-Leucine | 1124 mg |
| L-Lysine hydrochloride | 1110 mg |
| L-Methionine | 810 mg |
| L-Phenylalanine | 1089 mg |
| L-Threonine | 654 mg |
| L-Tryptophan | 189 mg |
| L-Valine | 876 mg |
| L-Histidine hydrochloride hydrate | 626 mg |
| L-Arginine hydrochloride | 1406 mg |
| L-Alanine | 1124 mg |
| Magnesium/potassium L-Aspartate | 1295 mg |
| Sodium L-Aspartate monohydrate | 1084 mg |
| L-Glutamine | 2415 mg |
| Glycine | 631 mg |
| L-Proline | 788 mg |
| L-Serine | 1449 mg |
| L-Tyrosine | 138 mg |
| Carbohydrate (dextrin) | 79.3 g (304 kcal) |
| Lipid (soybean oil) | 0.636 g (5 kcal) |
| Vitamin A | 810 IU |
| Vitamin D | 64.0 IU |
| Vitamin B-1 | 0.24 mg |
| Vitamin B-2 | 0.25 mg |
| Vitamin B-6 | 0.33 mg |
| Nicotinamide | 2.75 mg |
| Pantothenic acid | 1.38 mg |
| Folic acid | 55 μg |
| Vitamin B-12 | 0.88 μg |
| Vitamin C | 9.75 mg |
| Vitamin K | 11.3 μg |
| Vitamin E | 4.13 IU |
| Biotin | 48.8 μg |
| Choline | 10.7 mg |
| Na | 325 mg |
| K | 272 mg |
| Cl | 646 mg |
| Mg | 50.0 mg |
| Ca | 197 mg |
| P | 152 mg |
| Fe | 2.25 mg |
| I | 19.0 μg |
| Mn | 375 μg |
| Cu | 250 μg |
| Zn | 2.25 mg |

Na, sodium; K, potassium; Cl, chlorine; Mg, magnesium; Ca, calcium; P, phosphorus; Fe, iron; I, iodine; Mn, manganese; Cu, copper; Zn, zinc.
